# Data on taxa composition of freshwater zooplankton and meiobenthos across Arctic regions of Russia

**DOI:** 10.1016/j.dib.2021.107112

**Published:** 2021-05-15

**Authors:** Elena Fefilova, Olga Dubovskaya, Olga Kononova, Larisa Frolova, Ekaterina Abramova, Gulnara Nigamatzyanova

**Affiliations:** a Institute of Biology of Komi Science Centre of the Ural Branch of the Russian Academy of Sciences, Kommunisticheskaya 28, 167982, Syktyvkar, Russia; b Institute of Biophysics of Federal Research Center ``Krasnoyarsk Science Center'' of Siberian Branch of Russian Academy of Sciences, Akademgorodok 50/50, Krasnoyarsk 660036, Russia; c Siberian Federal University, Svobodny av. 79, 660041, Krasnoyarsk, Russia; d Institute of Geology and Petroleum Technologies, Kazan Federal University, Kremlyovskaya 18, 420008, Kazan, Russia; e Lena Delta Nature Reserve, Ak. Fedorova 28, 678400 Tiksi, Sakha Republic, Russia

**Keywords:** Arctic, Fresh waters, Rotifers, Cladocerans, Copepods, Zooplankton, Meiobenthos, Species list

## Abstract

We present the presence/absence species list (Table 1) of rotifer, cladoceran, and copepod (Calanoida, Harpacticoida, and Cyclopoida) fauna from seven Arctic regions of Russia (the Kola Peninsula, the Pechora River Delta, the Bolshezemelskaya tundra, the Polar Ural, the Putorana Plateau, the Lena River Delta, and the Indigirka River Basin) based on our own and literature data. Our own records were obtained by analyzing samples of zooplankton, meiobenthos, and two cores of bottom sediments (from the Kola Peninsula and the Bolshezemelskaya tundra lakes) that we collected once in July or August in 1992, 1995–2017. To supplement the list, we used relevant literature with periods of research from the 1960s to the 2010s. The list is almost identical to “Dataset 2: Zooplankton and Meiofauna across Arctic Regions of Russia”, which was analyzed but not published in [1]. The detailed analysis of this list revealed the specific composition of the aquatic fauna associated with the climatic and geographical factors [1]. The data provide information on the current state of biodiversity and species richness in Arctic fresh waters and can serve as the basis for monitoring these environments and predicting how they are likely to change in the future.

## Specifications Table

**Table:** 

Subject	Biodiversity
Specific subject area	Seven inland Arctic regions of European Russia and Siberia
Type of data	Table
How data were acquired	Equipment for field sampling: plankton nets with 82–100 μm mesh nylon, Ruttner samplers, Petersen dredge (sample area 0.025 m^2^), handle blade trawl with mesh size 230 μm or less, UWITEC piston corer, rod-operated half-tube corer (Russian peat corer: a 5 cm diameter, 100 cm long sampler). Microscopes: BIMAM R13-1, Leica DM 4000 B, PZO (Warszawa, Poland), Axioskop 40 (Carl Zeiss) microscope with a MC-3254R/MFG 3ccd color video camera (AVT-Horn, Aalen, Germany) and AxioVision 3.1 software, Carl Zeiss Axio Imager A2, Carl Zeiss Axio Lab.A1.
Data format	Filtered
Parameters for data collection	Data were obtained by analyzing zooplankton, zoobenthos and paleo samples (cores). The data showed species composition of rotifers, cladocerans, and copepods from chiefly lentic waterbodies of seven Arctic regions collected by us in 1992, 1995–2017 and complemented by literature data from the 1960s to the 2010s. Sampling and processing methods were identical throughout the data collection.
Description of data collection	Zooplankton samples were collected using plankton nets and Ruttner samplers. Benthic micro-crustaceans were sampled using the Petersen dredge and a handle blade trawl. Samples were fixed in the field. Subfossil cladocerans from two lakes were collected using the tube corers; paleo samples were processed in the laboratory. All samples were examined under light microscopes. Our own and literature data on sample composition were combined to compile the taxa presence/absence data set, where each column of the table corresponded to one region.
Data source location	Institutions: Institute of Biology of Komi Science Centre of the Ural Branch of the Russian Academy of Sciences; Institute of Biophysics of Federal Research Center “Krasnoyarsk Science Center” of Siberian Branch of Russian Academy of Sciences; Siberian Federal University; Institute of Geology and Petroleum Technologies, Kazan Federal University; Lena Delta Nature Reserve Regions: the Kola Peninsula, the Pechora River Delta, the Bolshezemelskaya tundra, the Polar Ural, the Putorana Plateau, the Lena River Delta, the Indigirka River Basin Country: Russia Latitude and longitude (and GPS coordinates, if possible) for collected samples/data: about 66.69 – 73.39 N, 33.62 – 147.52 E Primary data sources for some points ([2-8] in Table 1): A.N. Kruglova, Zooplankton of the Kola River (the Barents Sea Basin), Proceeding of Karelian Scientific Centre of RAS. 4 (2009), 85‒89. (In Russian). E.S. Makartseva, Zooplankton of lakes of different landscapes of Kola Peninsula, in: V.G. Drabkova, T.D. Slepukhina (Eds), Lakes of different landscapes of Kola Peninsula. Nauka, Leningrad. (1974), 143‒179. (In Russian). E.V. Borutskiy, Crustacea. Freshwater Harpacticoida, Fauna of U.S.S.R., Vol. 3(4). Academy of Sciences of USSR Press, Moscow, Leningrad. (1952), 425 pp. (In Russian). Hydrobiological study and fish agriculture application in lakes of the Extreme North, A.M. Gidalevich, M.T. Chernyakova (Eds), Nauka, Moscow. (1966) (In Russian). Flora and fauna of the waterbodies of the European North (case study of lakes of Bolshezemelskaya tundra), M.V. Getsen (Ed), Nauka, Leningrad. (1978), 178 pp. (In Russian).
	N.V. Vekhov, Life cycles of copepods of the Diaptomidae family (Crustacea, Calanoida) in waterbodies of the Subarctic Region in Europe, Ekologiya 3 (1988), 54-66. (In Russian). N.G. Sheveleva, Diversity of planktonic fauna of Putorana plateau water bodies, in: A.A. Romanov (Ed), Bird and animal communities of the Putorana Plateau: studies and conservation, Rosselhozakademia Press, Moscow. (2006), 239‒251. (In Russian).
Data accessibility	With the article Data on taxa composition of freshwater zooplankton and meiobenthos across Arctic regions of Russia Repository name: Mendeley Data Direct URL to data: http://dx.doi.org/10.17632/45tbyx9r3n.2 Fefilova, Elena; Dubovskaya, Olga; Kononova, Olga; Frolova, Larisa; Abramova, Ekaterina; Nigamatzyanova, Gulnara (2021), “The list of rotifer and micro-crustacean taxa identified in the inland waters of the regions of Russian Arctic ”, Mendeley Data, V2, doi:10.17632/45tbyx9r3n.3 http://dx.doi.org/10.17632/45tbyx9r3n.3 license CC BY 4.0
Related research article	E. Fefilova, O. Dubovskaya, L. Frolova, E. Abramova, O. Kononova, G. Nigamatzyanova, I. Zuev, E. Kochanova, Biogeographic patterns of planktonic and meiobenthic fauna diversity in inland waters of the Russian Arctic. Freshwater Biology (2020) 00:1‒17. https://doi.org/10.1111/fwb.13624

## Value of the Data

• The data provide information on the *current state of biodiversity* and *species richness* in fresh waters of seven Arctic regions of Russia and can serve as the basis for *monitoring* these environments. They are related to the research article: [1] (Fefilova et al.), and are important for better understanding of the analyses performed in [1].
• The data are valuable for scientists who study *biogeography* of freshwater micro-invertebrates. They may be used in applied sciences for estimation of regional resources for *nature-conservation measures*.
• These data are valuable for future analysis, i.e. for detailed and in-depth analysis of the distribution of certain species or groups (Rotifera, Cladocera, Copepoda), analysis of their range, etc.
• It is believed that the fauna of aquatic invertebrates in the Russian part of the Arctic has been poorly studied. This opinion is also supported by the limited access to raw data (mostly unpublished). Thus, these data enrich the fundamental knowledge about the composition of zooplankton and meiobenthos in the high latitudes of Russia.

## 1 Data Description

The presence/absence list of micro-invertebrate taxa from planktonic and benthic samples collected in inland continental waterbodies of seven regions in the Russian Arctic is provided in Supplementry Table 1. The Table contains a compiled list of the lower-rank taxa of micro-invertebrates (Rotifera, Cladocera and Copepoda) for the regions, which summarizes taxa from our own studies and additional species from literature marked by the number of Reference. This is freshwater fauna of chiefly lentic inland waters, based on modern taxonomy of the groups under study. Each column of Table 1 corresponds to one region: the Kola Peninsula (Region I), the Pechora River Delta (Region II), the Bolshezemelskaya tundra (Region III), the Polar Ural (Region IV), the Putorana Plateau (Region V), the Lena River Delta (Region VI), and the Indigirka River Basin (Region VII). The detailed analysis of the list of taxa is given in [1], where it is called “Dataset 2: Zooplankton and Meiofauna across Arctic Regions of Russia”. Here we publish this Dataset for the first time (Table 1). The number of species (species richness) in this Table 1 is equal to the number of species in Table 1 in [1] from Dataset 2 except the number of Cyclopoida species in Region II (15 versus 14, respectively), for which one species, *Thermocyclops oithonoides*, was omitted in Dataset 2. Note that this list is not suitable for analyzing temporal trends in species composition, they have been analyzed in [1] in separate regions, for example, III and VI.

## 2 Experimental Design, Materials and Methods

### 2.1 Data sources and study design

Our study area encompasses seven inland Arctic regions of Russia: the Kola Peninsula (Region I), the Pechora River Delta (Region II), the Bolshezemelskaya tundra (Region III), the Polar Ural (Region IV), the Putorana Plateau (Region V), the Lena River Delta (Region VI), and the Indigirka River Basin (Region VII), covering a longitudinal distance of 4,800 km, from the west to the east (about 33.62 – 147.52 E). All regions are situated north of the Arctic Circle (about 66.69 – 73.39 N).

The list of species of zooplankton and meiofauna contains our own findings and literature data chiefly for the period from the 1960s to the 2010s (1960-2017). Our own findings were obtained by analyzing samples of zooplankton, meiobenthos, and two cores of bottom sediments (from Regions I, Lake Antyuh-Lambina, and III, Lake Kharbey) that we collected once in summer (in July or August).

Specifically, the data for Region I included our sediment core information from one lake (Antyuh-Lambina) obtained in 2015 and lists of the species of zooplankton and benthic micro-crustaceans in waterbodies on the Kola Peninsula [2], [3], [4]. Data set for Region II included our own list of planktonic and benthic micro-fauna species of the Pechora River Delta based on 60 plankton samples collected in 2016 and 2017. For Region III, we used our data on more than 55 lentic waterbodies (lakes, ponds and pools), previously partly published in [9], [10], our unpublished data on lentic and lotic waters, and lists of planktonic and benthic micro-fauna of the Bolshezemelskaya tundra provided by other authors [5], [6], [7], for the entire period of 1960–2013; the total number of samples was more than 360 planktonic and more than 400 benthic ones. Our sediment core information from one lake of Region III (Lake Kharbey) obtained in 2012 was also included in the species list. Region IV comprised only our own list of zooplankton and meiofauna species from 9 lakes located on the western slope of the Ural Mountains. They were studied in 2003 (2 lakes), 2010 (1 lake), and 2013 (6 lakes); 21 plankton and 23 benthos samples were taken. For Region V, we provided our list of zooplankton species from 38 waterbodies (lakes, including deep and large lakes – Lama, Glubokoe, and Kutaramakan, and ponds), from which some parts were previously published [10], [11], [12], with additional species from the list of plankton fauna for lakes and reservoirs of the Putorana Plateau compiled by [8]. All 38 waterbodies were sampled during 2001–2004, and 4 of 38 again in 2011; the total number of samples was 94. Data [8] embraced the period from the 1960s to the 2000s. In Region VI, we used our plankton fauna list from 20 waterbodies (lakes and ponds) sampled in 1995, 1996 (4 waterbodies), during 2000–2017 (14 waterbodies) and 2014–2016 (2 lakes), which had been partly published previously, in [13]. In total, 558 plankton samples from this region were analyzed. For Region VII, we only used our own data on zooplankton composition in one lake (Suturuoha) and 27 polygonal ponds in the lower reaches of the Indigirka River. From the Suturuoha Lake, 16 plankton samples were collected in 2015; from ponds of this region 27 plankton samples (1 per waterbody) were taken in 2011. Thus, minimal sampling effort was rather high, namely, 44 samples in Region IV and 43 samples in Region VII; in Region III and Region VI sampling effort was maximal.

### 2.2 Field and laboratory methods

Zooplankton samples (for all published and unpublished data sets) were collected using plankton nets or Ruttner samplers with subsequent filtration through 82–100 μm mesh nylon nets. Similarly, all benthic micro-crustaceans in fine sediments were sampled using the Petersen dredge (sampling area 0.025 m^2^), while those on rocky bottoms and from shallow depths were collected by a handle blade trawl or net with mesh size ≤230 μm. Samples were preserved in 90–96% ethanol or 4% formaldehyde (in the field) and examined under light microscopes in the laboratory.

For the paleoecological study of cladocerans from a Bolshezemelskaya tundra lake (Lake Kharbey), a short (25 cm) sediment core was collected using a UWITEC piston corer. From a Kola Peninsula lake, a sediment core was collected with a rod-operated half-tube corer (Russian peat corer: a 5 cm diameter, 100 cm long sampler). For subfossil Cladocera analysis, the cores were divided into 1–2 cm subsamples, each subsample was dissolved in 10% KOH and heated to 75 °C for 30 min. The resulting suspension was sequentially sieved through 63 µm sieves for Lake Kharbey and 50 µm for Lake Antyukh-Lambina. All subsamples were examined under a light microscope in laboratory.

### 2.3 Taxonomic analysis

All species within Rotifera, Cladocera, and Copepoda were identified in zooplankton samples, while only Cladocera and Copepoda were identified in benthic samples. For identification of species and higher-level taxa, we used both standard keys and data reported in modern studies specifically addressing the taxonomy of these groups. Species that actually represented groups of related species were identified as species (s. lat.) according to descriptions in taxonomic keys and studies [14], [15], [16], [17].

## Ethics Statement

The work did not involve the use of human subjects, animal experiments and data collected from social media platforms.

## CRediT Author Statement

**Elena Fefilova:** Conceptualization, Data curation, Formal analysis, Resources, Writing - original draft, Writing - review & editing; **Olga Dubovskaya:** Data curation, Writing - original draft_ Writing - review & editing; **Olga Kononova:** Data curation; **Larisa Frolova:** Data curation; **Ekaterina Abramova:** Data curation; **Gulnara Nigamatzyanova:** Data curation.

## Declaration of Competing Interest

The authors declare that they have no known competing financial interests or personal relationships which have or could be perceived to have influenced the work reported in this article.

## Appendix Supplementary materials

Image, application 1

## Footnotes

Supplementary material associated with this article can be found in the online version at doi:10.1016/j.dib.2021.107112.
